# Botulinum toxin A increases allograft tolerance in an experimental transplantation model: a preliminary study

**DOI:** 10.1042/BSR20171721

**Published:** 2018-03-21

**Authors:** Yun Joo Park, Jang Won Lee, Yosep Chong, Tae Hwan Park

**Affiliations:** 1 Department of Radiology, Hallym University College of Medicine, Hallym University Sacred Heart Hospital, 22, Gwanpyeong-ro 170 beon-gil, Dongan-gu, Anyang 14068, Gyeonggi-do, Republic of Korea; 2 Department of Plastic and Reconstructive Surgery, CHA Bundang Medical Center, CHA University, 59 Yatap-ro, Bundang-gu, Seongnam, Gyeonggi-do 13496, Republic of Korea; 3 Department of Hospital Pathology, College of Medicine, The Catholic University of Korea, Seoul, Republic of Korea

**Keywords:** botulinum toxin A, rejection, skin, transplantation, tolerance

## Abstract

Identifying novel and safe immunosuppressants is of crucial importance. Recently, there have been several studies revealing that botulinum toxin A (BoTA) significantly alleviates ischemia–reperfusion injuries. Emerging evidence shows that ischemia–reperfusion injuries contribute to innate immune activation, promoting rejection, and inhibiting tolerance. Therefore, we hypothesized that a pretreatment with BoTA might decrease allograft rejection in a rat transplantation model. Twenty-four Lewis (LEW) rats were randomly assigned into two groups consisting of 12 rats each, depending on whether skin allograft was performed after pretreatment with BoTA (BoTA group) or with normal saline (control group). The experimental group was pretreated with a subcutaneous injection of BoTA (10 IU), while the control group was pretreated with normal saline 5 days prior to surgery. The donor Brown–Norway (BN) rat dorsal skin was subsequently grafted to the recipient LEW rats. The recipient wounds, measuring 2 cm × 2 cm, were made via dorsal skin excision through the panniculus carnosus. The donor skins of the same dimensions were obtained and transplanted on to the wounds and sutured with 4-0 nylon sutures. Mean graft survival time was measured in both groups. Quantitative reverse-transcriptase PCR and Western blotting were performed to evaluate the gene/protein expression of CD4 and VEGF. The mean graft survival time in the BoTA group was significantly longer than that of the control group (*P*=0.004). The relative mRNA and protein expression of CD4 was significantly lower in the BoTA group (*P*<0.001), while the relative mRNA and protein expression of VEGF was significantly higher in the BoTA group (*P*<0.001). In conclusion, our results show that BoTA prolongs the survival of skin allografts in a rat transplantation model.

## Introduction

The use of known allograft immunosuppressants inevitably causes systemic complications. Cyclosporine A (CsA), tacrolimus (FK 506), and sirolimus are amongst those that are conventionally used and investigated. These have significantly improved allograft survival but show side effects associated with long-term usage. Therefore, identifying novel and safe immunosuppressants is of great importance to clinical practice. Research suggests that skin has the most pronounced immunogenicity, resulting in allograft rejection following transplantation [1–3]. Therefore, skin transplantation is used as a reliable model of allograft rejection.

Botulinum toxin A (BoTA) is a widely used material in various clinical situations for aesthetic and reconstructive purposes. Recently, there have been several studies revealing that BoTA significantly alleviates ischemia–reperfusion injury [14,15]. Additionally, even though the evidence regarding ischemia–reperfusion injury in vascularized tissue allotransplantation remains unclear, emerging data show that it contribute to innate immune activation, promoting rejection, and inhibiting tolerance [4,5]. Therefore, any strategy that minimizes the effect of ischemia–reperfusion injuries might aid in reducing allograft rejection. In the present study, we hypothesized that BoTA might protect from skin allograft rejection using an MHC full mismatch rat model.

## Methods

Twenty-four 8-week-old male Brown–Norway (BN) rats were used as donors, and 24 Lewis (LEW) rats weighing 240–300 g were used as recipients in the transplant procedure. All animal protocols used in the present study were approved by the Institutional Animal Care and Use Committee of Konkuk University and were carried out in accordance with the approved guidelines. The 24 LEW rats were randomly assigned into two groups consisting of 12 rats each, depending on whether the skin allograft was performed after pretreatment with BoTA (BoTA group) or with normal saline (control group).

The animals were initially administered general anesthesia with 5% isoflurane (Aerane®, Ilsung Pharmaceuticals, Seoul, South Korea) in an induction chamber, and were subsequently maintained under 1.5% isoflurane using a nasal cone. A 2 cm × 2 cm sized rectangular shaped flap was marked on each rat’s abdomen after shaving the ventral hair.

Five days prior to flap elevation, 100 IU vials of lyophilized BoTA were reconstituted in 10 ml normal saline, to obtain a final concentration of 10 IU/ml. The BoTA group was then pretreated with 10 IU of BoTA (BOTOX®, Allergan, Irvine, CA, U.S.A.) injected subdermally, while the control group was administered 0.8 ml normal saline. The recipient wounds, measuring 2 cm × 2 cm, were made by dorsal skin excision through the panniculus carnosus. The donor skins of the same dimension were harvested and transplanted on to the wounds and sutured with 4-0 nylon sutures.

The grafts were covered with a sterile bactericidal gauge, and each animal was housed in a separate cage after the grafting. All data generated or analyzed during the present study are included in this article.

### Skin allograft survival

Allografts were observed and photographed regularly until the rats were killed; the photographs were then imported into a computer and analyzed using the ImagePro Plus 6.0 (Media Cybernetics, Inc., Bethesda, U.S.A.) picture analysis software. Graft necrosis was grossly determined based on the graft’s appearance, color, texture, and absence of bleeding when cut with a scalpel. Necrosis rates were measured and >90% necrosis of the skin allograft was defined as rejection. The mean survival time (MST) of skin grafts in both groups was observed in 16 rats (8 rats from the BoTA group and 8 rats from the control group); the remaining 8 rats were used to examine gene and protein expressions.

### Gene expression

At 10 days post operation, the full thickness total skin graft was harvested from each of the randomly selected eight rats (four rats from BoTA group and four rats from control group) before killing, snap-frozen in liquid nitrogen, and then stored at –80°C. Quantitative reverse-transcriptase PCR (qRT-PCR) was performed using primers for the *CD4* and *VEGF* genes, while glyceraldehyde-3-phosphate dehydrogenase (*GAPDH*), a housekeeping gene, was used as control (Table 1). Cells from the collected tissue were homogenized in 1 ml TRIzol reagent (T9424, Sigma–Aldrich, South Korea) at room temperature for 5 min. After 0.2 ml chloroform was added, the tubes were shaken well for 15 s and left at room temperature for 3 min. The solution was then centrifuged at 11500 rpm for 15 min at 4°C, and the aqueous portion was transferred to fresh tubes. RNA precipitation was done by adding 0.5 ml isopropanol. These tubes were then mixed by inversion, incubated at room temperature for 10 min, and centrifuged at 11500 rpm for 10 min at 4°C. After the supernatant was removed and mixed with –20°C ethanol, the solution was centrifuged at 9500 rpm for 5 min at 4°C. The supernatant was quickly removed, and the precipitate was dried under sterile conditions. The RNA pellet was re-dissolved in 40 µl of diethylpyrocarbonate (DEPC) water. The samples’ optical densities were measured at a wavelength of 260 nm using a NanoDrop® spectrophotometer (DaeMyung Science, South Korea).

**Table 1:** Primer sequences for *CD4, VEGF*, and *GAPDH* with the qRT-PCR experimental conditions

Gene	Primer	Cycle	bp	
*CD4*	Forward (5′–3′)	TATAAGAGTGAGGGGGAGTC	50	113
	Reverse (5′–3′)	CAGGACTGGGAAGAAGGAGC		
*VEGF*	Forward (5′–3′)	CTGCTGTACCTCCACCATGC	50	300
	Reverse (5′–3′)	CTGGCTTTGGTGAGGTTTGA		
*GAPDH*	Forward (5′–3′)	GACAAGATGGTGAAGGTCGG	50	234
	Reverse (5′–3′)	CTGGAAGATGGTGATGGGTTT		

Gene expression was quantitated by real-time PCR using the LightCycler® 480 II (Roche, South Korea). The primer sequences of the genes under study were synthesized by Bioneer Corp. (South Korea) and are listed in Table 2. The reaction mixture contained 20 ng cDNA, 10 µl 2× SYBR Green I Master Mix, 100 pmol F primer, 100 pmol R primer, and distilled water added to a volume of 20 µl. The reaction conditions were as follows: 10 min at 50°C for reverse transcription, 5 min at 95°C for initial activation, 50 cycles of 15 s at 95°C for denaturation, 10 s at 60°C for annealing, and 10 s at 72°C for elongation. Product specificity of the PCR was confirmed using a dissociation curve. Each run included a non-template control. Fluorescence was detected at 72°C. The results were evaluated using Light Cycler® 480 analysis software. *GAPDH* served as a reference housekeeping gene. All the reactions were performed in triplicate.

**Table 2:** Primary and secondary antibodies used in the experiments and their dilution factors

**Antibody**	**Dilution factor**
Primary	
Anti-B-actin (mouse)	1:5000
Anti-CD4 (mouse)	1:1000
Anti-VEGF (rabbit)	1:200
Secondary	
Anti-mouse	1:10000
Anti-rabbit	1:10000

### Protein expression

On postoperative day 10, a full thickness total skin graft was harvested and immediately snap-frozen in liquid nitrogen and stored at –80°C in RIPA buffer (50 mmol/l Tris/HCl (pH 7.6), 150 mmol/l NaCl, 1% NP40, 0.5% sodium deoxycholate, 0.1% SDS) supplemented with a protease inhibitor cocktail. The solution was left at –4°C for 10 min and then centrifuged at 4°C at 14000 rpm for 30 min; the aqueous solution was subsequently transferred to a fresh tube.

The solutions were boiled in SDS sample buffer, resolved by SDS/PAGE, and electrically transferred on to a nitrocellulose membrane (Bio–Rad Laboratories, Germany). Subsequently, Western blotting was performed with the appropriate primary antibodies and horseradish peroxidase–conjugated secondary antibodies (Table 2). Antibody–protein complexes were visualized using ImageQuant LAS 4000 (GE Healthcare, Pittsburgh, PA, U.S.A.). Signal intensities of the bands were quantitated using the analysis software Multi Gauge v3.1 (Fujifilm).

### Statistical analysis

The results are expressed as means ± S.D. Student’s *t* test was used for the comparison of MST, relative mRNA expression, and relative protein expression. A *P*-value of less than 0.05 was considered statistically significant.

## Results

### Skin allograft survival

Graft MST of the BoTA group was significantly longer ((*P*=0.008), 15.50 ± 0.93 days) than that of the control group (14.13 ± 0.83 days). Postoperative infections in the skin grafts were not observed in either group.

**Figure 1:**
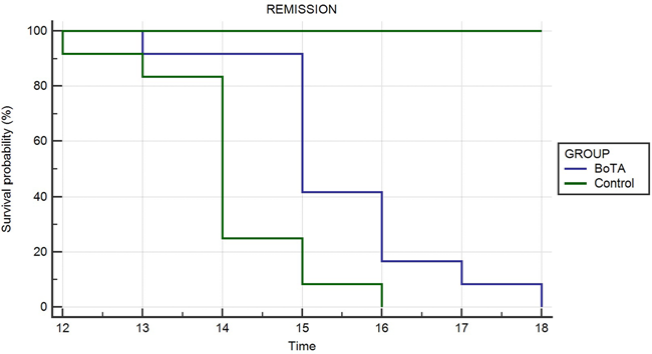
Survival times of skin allografts Kaplan–Meier survival curves show longer survival time in the BoTA group than the control group.

**Figure 2:**
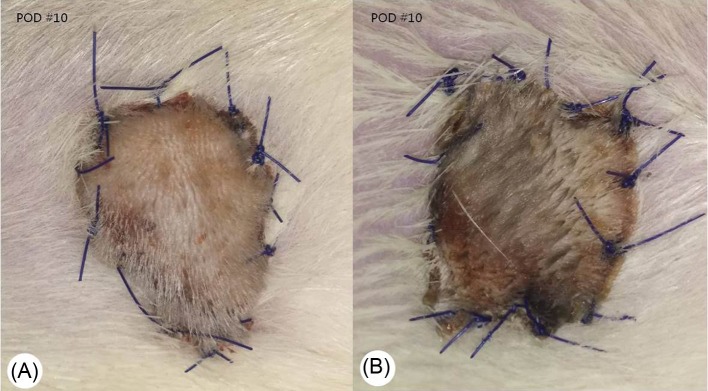
Gross findings of the skin allografts in representative animals from both groups Photographs were taken 7 days after transplantation and rats treated with BoTA showed better allograft survival compared with the control group rats on postoperative day 10.

The above-mentioned flap survival curves using Kaplan–Meier plot are presented in Figure 1 and the representative gross images at postoperative day 10 are presented in Figure 2.

### Gene expressions

**Figure 3:**
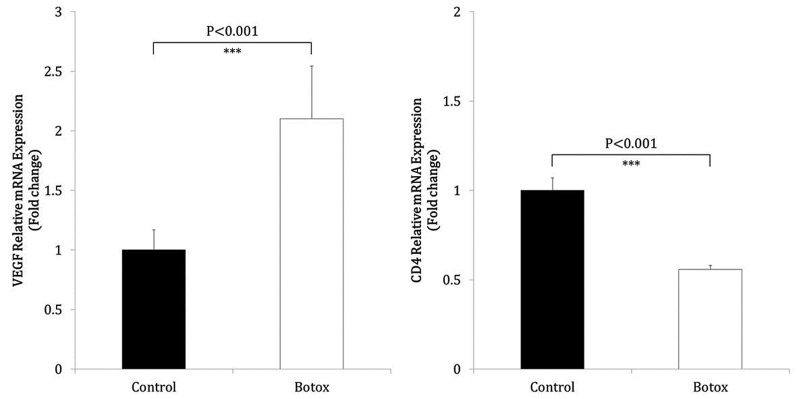
Effects of BoTA on the mRNA expression of *VEGF* and *CD4* BoTA treatment significantly increased the mRNA expression of *VEGF* on postoperative day 10 after skin transplantation compared with the control group (*n*=8, *P*<0.001). In contrast, BoTA treatment significantly inhibited the mRNA expression of *CD4* on postoperative day 10 after skin transplantation compared with the control group (*n*=8, *P*<0.001).

The relative mRNA expression of *CD4* was significantly lower in the BoTA group than in the control group (*P*<0.001, Figure 3). The relative mRNA expression of *VEGF* was significantly higher in the BoTA group than in the control group (*P*<0.001, Figure 3).

### Protein expression

**Figure 4:**
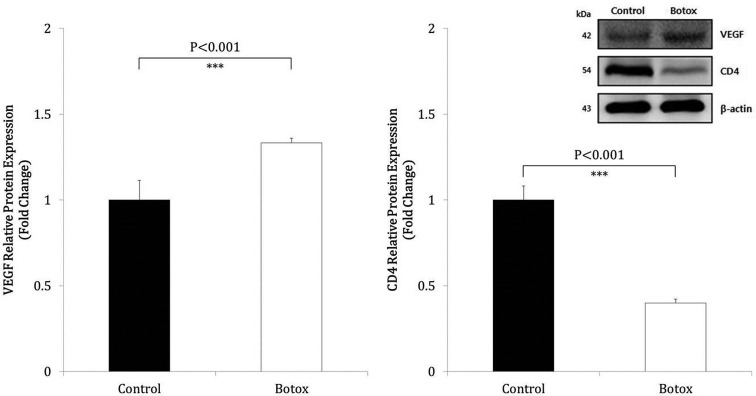
Effects of BoTA on the expression of VEGF and CD4 proteins BoTA treatment significantly increased the expression of VEGF protein on postoperative day 10 after skin transplantation compared with the control group (*n*=8, *P*<0.001). In contrast, BoTA treatment significantly inhibited the expression of CD4 protein on postoperative day 10 after skin transplantation compared with the control group (*n*=8, *P*<0.001). Representative Western blots of CD4 and VEGF with β-actin as loading controls are also shown.

The relative protein expression of CD4 was significantly lower in the BoTA group than the control group (*P*<0.001, Figure 4). On the contrary, expression of VEGF was significantly higher in the BoTA group than the control group (*P*<0.001, Figure 4). Representative Western blots of CD4 and VEGF of β-actin are also shown in Figure 4.

## Discussion

In the current study, we observed the protective effect of BoTA on allograft rejection for the first time and discussed its related mechanisms.

The skin allograft model is known to be the most potent and convenient experimental model for the study of immunologically mediated tissue rejection. To counteract rejection responses, various studies have examined its validity for the assessment of allograft rejection [1,2,3,4,5,6]. Our present study confirms that BoTA promotes skin allograft survival and reduces the occurrence of acute rejection. Unlike other immune modulators, BoTA is safe, widely used material for aesthetic and reconstructive purposes. Prior to the current study, we determined the appropriate dosage of our BoTA through a pilot study with different dosages. The dose of 10 U of BoTA seemed to be effective and harmless without systemic effects. This is in line with a previous study by Park et al. [7] involving 24 rats pretreated with 20 U of BoTA; the result was increased flap survival after 10 days.

**Figure 5:**
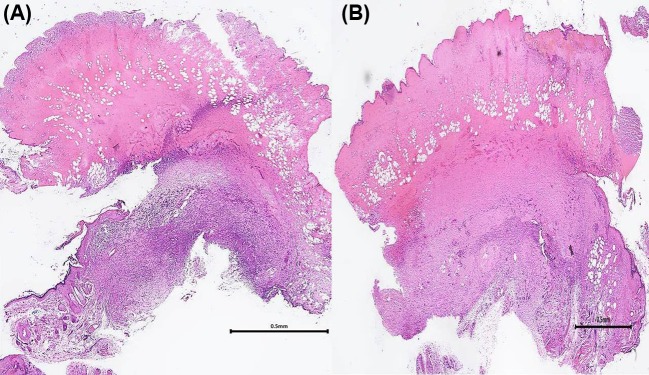
Histological findings of the representative slides of the BoTA and control groups (H&E stain, ×12.5) There was more stromal hemorrhage, fibrosis, and inflammatory cell infiltration in general in the control group (**B**) than that in the BoTA group (**A**).

**Figure 6:**
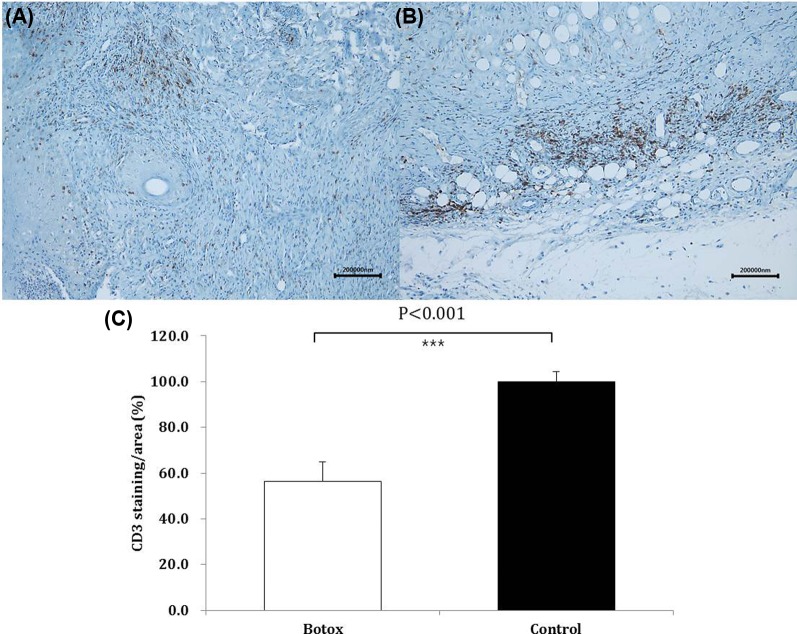
Effect of BoTA on skin allograft pathology CD3 staining in BoTA group I (**A**) and in the control group (**B**). Representative skin allografts in the control groups revealed relatively higher degree of skin rejection, accompanied by mixed lymphocyte infiltration compared with the BoTA group. (**C**) A semiquantitative analysis demonstrated that CD3 expression was significantly lower in the experimental groups compared with the control group.

Inflammatory cells, including macrophages and lymphocytes, participate in allograft rejection by infiltrating into skin grafts and releasing various cytokines and mediators. Inflammatory cell infiltration has thus been recognized as an important factor in allograft rejection [8]. In addition, leukocyte recruitment to the allograft during the rejection cascade is reportedly similar to that of inflammatory reponses [5]. For this reason, interventions inhibiting inflammatory cell infiltration may also work in prolonging allograft survival by reducing T cell and macrophage infiltration as well as decreasing the expression of mediators and cytokines. The anti-inflammatory effects of BoTA have been intensively examined in clinical studies. Many studies have shown the therapeutic effect of intravesical BoTA on bladder pain syndrome/interstitial cystitis. A recent study by Jhang et al. showed that a BoTA injection could induce peripheral desensitization, reduce chronic bladder inflammation, and decrease apoptotic signal molecules in the urothelium in bladder pain syndrome/interstitial cystitis [9].In the current study, we performed immunohistochemical staining using CD3 antibodies to test the effect of BoTA on T-lymphocyte infiltration. Our results show that treatment with BoTA can significantly attenuate inflammatory cell infiltration in pathologic level at postoperative day 10 (Figure 5). CD3 expression was semiquantitatively analyzed using ImageJ analysis software (National Institutes of Health, Bethesda, MD, U.S.A.). CD3 expression was found to be significantly lower in the experimental group than in the control group (Figure 6). In addition to the pathologic findings, the present experiment demonstrated that BoTA could significantly down-regulate CD4 expression of at both transcriptional and post-translational levels, which explains the reduction in skin graft inflammatory cell infiltration after pretreating with BoTA.

A recent study showed that BoTA increases the angiogenesis HIF-1α/VEGF signaling in a dependent manner [10]. This finding is also evidenced by our results, which showed an increase in VEGF expression at both transcriptional and post-translational levels in the BoTA group. Despite this increase, the BoTA group had a significantly decreased CD4 expression. This finding is of particular interest as many classic immunosuppressive agents act on immune rejection by inhibiting angiogenesis. This point to the possibility of BoTA having ideal properties for increasing allograft tolerance by stimulating VEGF expression, a marker of angiogenesis, and inhibiting CD4 expression which is a surface marker of immune cells such as T helper cells, monocytes, macrophages, and dendritic cells.

In the present study, 5 days prior to skin transplantation, the recipients were treated with BoTA (10 IU) considering that the maximal effects of BoTA on tissues are known to occur approximately 2 weeks after injection. Previous studies used multiple injections of immune modulators with regular intervals [11]. Compared with other protocols, our is very cost-effective and causes minimal discomfort when applied to clinical practice.

Developments in the field of organ and tissue transplantation have accelerated remarkably since the human MHC was discovered in 1967. Our current experiment model adopts strong rejection model using full MHC mismatch donor BN and recipient LEW rats. Therefore, our experiment has merit in simulating strong rejection model. One disadvantage of the used model is the possible misinterpretation of gross findings due to the dark skin color of BN rats. Hence, in this model, molecular data are of particular value to measure the protective effect of any immunosuppressants.

Amongst numerous molecules believed to be involved in skin allotransplantation, we selected the *CD4* and *VEGF* in terms of gene and protein expressions. In skin-transplanted animal, indirect alloresponses were invariably directed to a single or a few dominant determinants on donor MHC antigen. Recipient antigen-presenting cells (APCs) process and present donor MHC molecules, in the form of peptides, eliciting a T-cell response by *CD4^+^* T cells. Hence, *CD4* was used as a key molecule involved in our experiment [12]. Additionally, we selected *VEGF* as another crucial molecule. *VEGF* is generally considered as one of the most important molecule involved in a successful skin graft uptake. Based on our previous patient derived tumor xenograft model, increased *VEGF* expression is one of the meaningful markers to predict successful transplantation of humanized tumor tissue into the experimental immune-preserved animals [13].

Given the preliminary nature of the present study, we used a single BoTA injection, instead of testing optimal dosage with regular injections. We believe more injections at a regular interval would yield better outcomes in terms of allograft skin survival. Hence, future studies should be done to further explore the mechanisms of allograft tolerance and optimal dosage and route. Second, as we did not perform a positive control experiment simultaneously, we cannot objectively compare the degree of allograft tolerance of BoTA with conventional immunosuppressants such as CsA and FK 506. Finally, we did not check cytokine levels, which play a crucial role in transplant immunology. Thus, further efforts are warranted to investigate the immunosuppressive effect of BoTA.

## Conclusion

In conclusion, our results show that BoTA prolongs allogeneic skin graft survival at both transcriptional and post-translational levels in a rat skin transplantation model.

## Abbreviations

BN: Brown–Norway
BoTA: botulinum toxin A
CsA: cyclosporine A
FK 506: tacrolimus
GAPDH: glyceraldehyde-3-phosphate dehydrogenase
H&E: hematoxylin and eosin
HIF-1α: hypoxia-inducible factor 1-α
LEW: Lewis
MST: mean survival time
VEGF: vascular endothelial growth factor
